# Biomimetic Hydroxyapatite Growth on Functionalized Surfaces of Ti-6Al-4V and Ti-Zr-Nb Alloys

**DOI:** 10.1186/s11671-015-1017-x

**Published:** 2015-08-22

**Authors:** Ie V. Pylypchuk, A. L. Petranovskaya, P. P. Gorbyk, A. M. Korduban, P. E. Markovsky, O. M. Ivasishin

**Affiliations:** Chuiko Institute of Surface Chemistry of National Academy of Sciences of Ukraine, 17 General Naumov Str., Kyiv, 03164 Ukraine; G.V. Kurdyumov Institute for Metal Physics, National Academy of Sciences of Ukraine, Academician Vernadsky blvd., 36, 03680 Kyiv-142, Ukraine

**Keywords:** Biomimetic hydroxyapatite, Biocompatible titanium alloys, Ti-6Al-4V, Ti-Zr-Nb, Simulated body fluid, 82.33.Pt, 82.65.+r, 82.80.Pv, 68.35.-p, 81.07.Pr, 81.16.Dn

## Abstract

A biomimetic approach for coating titanium-containing alloys with hydroxyapatite (HA) is reported in the article. Two types of Ti-containing alloys were chosen as an object for coating: Ti-6Al-4V (recommended for orthopedic application) and a novel highly biocompatible Ti-Zr-Nb alloy, with good mechanical compatibility due to a modulus that is more close to that of human bones (E ≈ 50 GPa instead of 110 GPa in Ti-6Al-4V). Coating process was carried out in a 10×-concentrated simulated body fluid (SBF)—synthetic analog of human body plasma. The effect of oxidized and carboxylated alloy surface on formation of biomimetic hydroxyapatite has been studied. By XRD, we found influence of thermal conditions on HA crystal formation and size. SEM images and Fourier transform infrared confirmed that hydroxyapatite with different morphology, crystallinity, and Ca/P ratio formed on metallic surfaces. X-ray photoelectron spectroscopy showed that in the Ti-6AL-4V sample the observed Ca/P ratio reach 0.97, whereas in the Ti-Zr-Nb sample the observed Ca/P ratio reach 1.15.

## Background

Titanium alloys are the most widely used material for the manufacturing of implants. This is due to their high inertness to biological environments and close-to-human-bone mechanical properties as compared to stainless steel and other alloys employed in medical practice [1]. However, after implantation with titanium alloy implants, some problems in conjunction with bone and other tissues have arisen in individual cases. That is why the possibility of applying to the surface of titanium implants more biocompatible coatings is still very important. One of the most preferred ways to solve this problem is the coating with a hydroxyapatite.

A coating of hydroxyapatite (HA) layer can be deposited onto the metal alloy to assist the osseointegration of these implants with surrounding tissues [2]. The main reason for using HA coating on metallic substrates is to keep the mechanical properties of the metal such as load-bearing capability and, at the same time, to take advantage of the coating’s chemical similarity and biocompatibility with the human bone [3].

Various methods have been reported to produce nanosized apatite crystals, including chemical precipitation [3], solid-state reaction [4], hydrothermal synthesis [5], sol-gel synthesis [6], emulsion technique [7], and the laser sintering approach [8], whereas most routine approaches ca not control the crystal morphology and size precisely. Unfortunately, subsequent heat treatment at high temperature results in cracking and poor bond strength between the hydroxyapatite coating and the metal substrate. Further, an HA coating of high crystallinity, which is desirable for optimal biocompatibility, could not be achieved through these methods [8]. Much attention is paid to biomimetic synthesis because it can prepare nanosized apatite crystals with controllable size [9].

To simulate the natural properties of HA and its formation on a titanium substrate, in the last decade, the biomimetic method is commonly used, which consists in the creation of nucleation sites on the metal surface by modifying its surface by functional groups and furthers the process of mineralization in the simulated body fluids (SBF) equal by chemical composition to human plasma. In vitro mineralization/crystallization of HA from SBF can be induced by the especially treated metal implant surfaces. Therefore, great attention has been paid to give the metal substrates a capability to induce HA formation by attaching functional groups onto their surfaces [9]. It was shown that the process of HA formation occurred on negatively charged surfaces that contained, for instance, –OH or –COOH groups [10] and lead to the formation of good crystalline HA with a Ca/P ratio close to 1.67.

Large attention is paid to the development of the new advanced alloys that combine high biological inertness and mechanical compatibility with bone tissues [11]. The latter is achieved by reducing the elastic modulus of the alloys to a value close to that of the bones (20–30 GPa). In addition, when choosing a low modulus material for medical applications, it is necessary to consider the following fundamental aspects. First, the chemical composition of the alloy should be selected so that both its base and the alloying elements be, on the one hand, nontoxic and, on the other hand, their choice facilitates a decrease in the interatomic binding forces in the alloy [12–15]. Second, in addition to achieving low modulus, a large strain recovery of the material should be attained, which can be done either by increasing the yield stress of the material by creating a favorable microstructure and phase composition or by the implementation of a reversible martensitic transformation [12–15].

Resentment, aseptic inflammation, the occurrence of connective tissue capsule around the implant, metal corrosion, the appearance of negative cardiovascular responses during endovascular interventions, the occurrence of restenosis, occlusion, and rigidity are generally the results of the organism’s reaction to a foreign body with different mechanical properties. Thus, after finding out problems of cytotoxicity of chemical elements, to the forefront comes the problem of obtaining medical materials with mechanical compatibility with living human tissues (bones, vessels, etc.). For mechanical compatibility with biological tissues, what are necessary are materials with a low elastic modulus (E), close to the characteristics of organic tissues. Thus, biocompatible metals has such elastic modulus values: in Zr ~ 95GPa, Ti ~ 110GPa, Hf ~ 135GPa, Pt ~ 150Gpa, whereas bone ~ 20-30 GPa.

Thus, implants, produced from alloys developed earlier, do not have the desired low modulus of elasticity, or a low modulus of elasticity of reversible deformation is observed only in the tensile and compressive stresses when they lose their desired properties.

To ensure the necessary mechanical compatibility of metallic materials, they must, in addition, have a reversible deformation, which is inherent to biological tissues.

Such unique properties can be achieved because of the formation of a biocompatible alloy with low elastic modulus based on zirconium-titanium-containing zirconium, titanium, niobium, and alloy components recently developed in the G.V. Kurdyumov Institute for Metal Physics (IMP) NAS of Ukraine.

This new alloy has the following main features: (i) excellent biocompatibility due to inclusion into its composition of only neutral chemical elements for the human body (contrary to Ti-6Al-4V which includes such relatively dangerous elements like aluminum and vanadium) and (ii) good mechanical compatibility due to low modulus more close to human bones (E ≈ 50 GPa instead of 110 GPa in Ti-6Al-4V), high reversible deformation (also close to numbers of bones—elastic elongation ≥2.9 %), and high tensile and fatigue strengths (≥1100 and 600 MPa) that ensures high reliability of implants during service period [12, 13]. Such unique properties were achieved as a result of the formation (by choice of the chemical composition) of mostly single-phase stable β condition with some inclusion of ω phase providing stable mechanical characteristics and geometrical dimensions of implants during long period of high-loaded cycled loading.

The main goal of this article is finding peculiarities of the HA formation processes on the new biocompatible alloy and its carboxylated surface.

## Methods

### Materials

Two alloys were chosen for coating by HA. The first of them was VT6 (or Ti-6Al-4V in the international designation), having the following composition: 6 (wt.)% Al, 4 % V, Ti-balance) which is still up to the present time the main material for the manufacture of a wide range of implants for the human body [12]. The second one is Zr-22 (at.)% Ti-18 Nb alloy recently developed in the G.V. Kurdyumov Institute for Metal Physics (IMP) NAS of Ukraine [14].

### Methods

*Specimens of* Ti-6Al-4V (VT6) for coating were cut into discs from a commercial rod having a diameter of 20 mm and then flattened out by grinding to 1-mm thickness. Zr-Ti-Nb alloy with the same diameter rod was produced in IMP via elemental powder approach [14, 16], and specimens were cut and grinded in the same manner as Ti-6Al-4V.

**Scheme 1:**
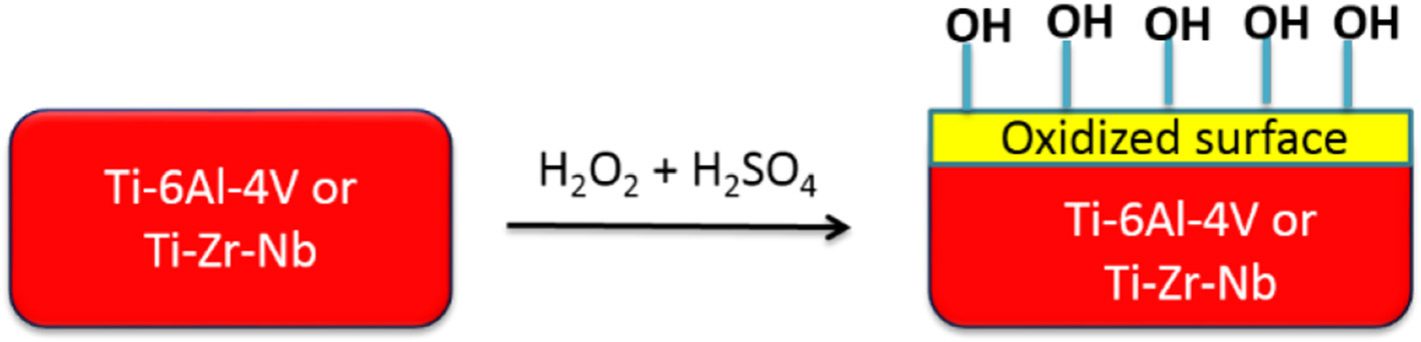
Oxidation/hydroxylation of alloy surface

*Pre-treatment* of titanium alloys was performed as follows. Firstly, alloy plates were mechanically cleaned then degreased with acetone and kept in an ultrasonic bath for 6–10 min to completely remove the organics. Next, the surface of samples was oxidized in the reaction mixture of H_2_O_2_ (30 %):H_2_SO_4_ (conc.) = 1:1 (by volume) with stirring for 10–15 min at room temperature. After preparation, the plates were washed with distilled water. After oxidation, we obtained Ti-6Al-4V–OH and Ti-Zr-Nb–OH samples (Scheme 1).

**Scheme 2:**
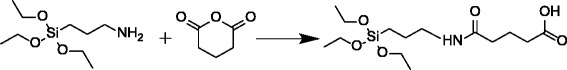
Synthesis of TESPCBA

*Triethoxysilylpropylcarbamoyl butanoic acid* (*TESPCBA*) was obtained by reacting γ-aminopropyltriethoxysilane with glutaric anhydride in anhydrous dimethylformamide (DMF) (Scheme 2).

**Scheme 3:**
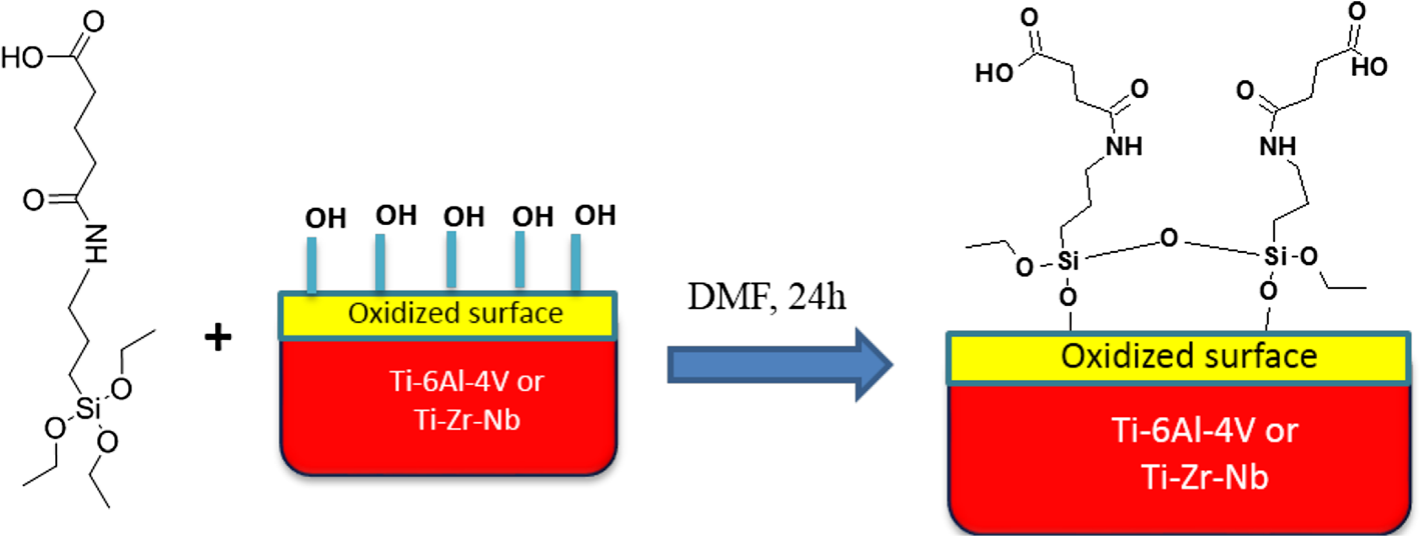
Surface modification of titanium by –COOH functional groups

*Synthesis of the reactive carboxyl groups* (Scheme 3) on the surface of titanium alloys was performed by modifying their oxidized surface by TESPCBA in DMF for 24 h.

Simulated body fluid solutions (Table 1) were prepared according to [17].

**Table 1:** Composition of human blood plasma, 10×-concentrated simulated body fluid (SBF) and Tas-SBF

Ion	Concentration (mM)		
	Human Body Plasma	SBF	Tas-SBF
Na^+^	142.0	142.0	142.0
K^+^	5.0	5.0	5.0
Mg^2+^	1.5	1.5	1.5
Ca^2+^	2.5	2.5	
**Cl** ^**−**^	**103.0**	**148.8**	**125.0**
**HCO** ^**3−**^	**27.0**	**4.2**	**27.0**
HPO_4_^2−^	1.0	1.0	1.0
SO_4_^2−^	0.5	0.5	0.5

bold numbers shows difference in ion concentrations for compared simulated body fluids

As a direct consequence, nucleation and precipitation of calcium phosphates from SBF are rather slow [18]. To get total surface coverage of a 10 × 10 × 1 mm titanium or titanium alloy substrate immersed into a 1.5× SBF solution, one typically needs to wait for 2 to 3 weeks, with frequent replenishment of the solution [17–19]. 10×-SBF was chosen to enhance the kinetics of coating deposition.

*X-ray diffraction analysis* (*XRD)* was performed using a DRON-UM1 diffractometer (Burevestnik, St. Petersburg) with Co K_α_ (*λ* = 0.17902 nm) radiation and graphite monochromator in reflected beam. XRD patterns of the samples were recorded over 2*θ* = 10–80° range*.*

*Fourier transform infrared spectroscopy* (*FTIR*) of the sample surface was performed by a Perkin Elmer, model 1720H. Diffusion reflectance spectra were collected.

*X-ray photoelectron spectra* of Са2p and P2p levels using Gauss-Newton method were mathematically decomposited into linked components with parameters ΔЕ_3/2–1/2_ = 1.0 eV and І_1/2_/І_3/2_ = 0.5, respectively. Their width at half maximum was amounted to ΔЕ = 1.4 eV in the case of the Са2p line and ΔЕ = 1.3 eV in the case of the Р2р line.

*Scanning electron microscopy (SEM)* performed at РЕМ-106 microscope, produced by “Electron” company located in Sumy, Ukraine.

## Results and discussion

**Fig. 1:**
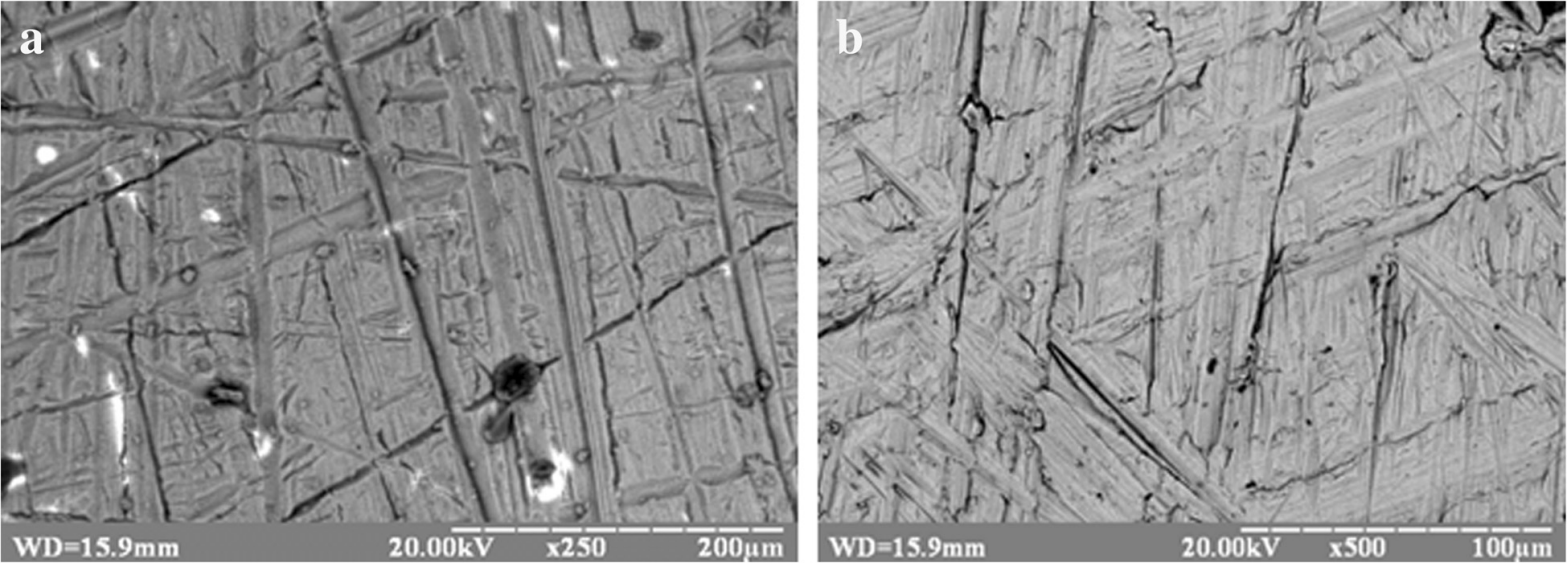
SEM images of Ti-6Al-4V (**a**) and Ti-Zr-Nb (**b**) alloy oxidized surfaces

Scanning electron microscopy (SEM) images of Ti-6Al-4V and Ti-Zr-Nb oxidized surfaces are presented in Fig. 1a, b, respectively. SEM defined the surface morphology as a rough microstructure with chaotic grooves (scratches). There, we can observe furrows that appeared after cleaning of the surface with sandpaper. The surface of oxidized Ti-6Al-4V alloy contains more local defects than Ti-Nb-Zr.

**Fig. 2:**
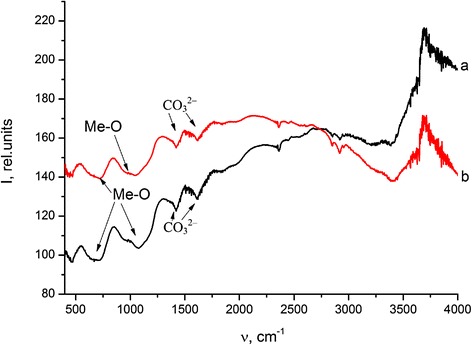
FTIR spectra of initial Ti-6Al-4V (*a*) and Ti-Zr-Nb (*b*) alloy surfaces

Initial samples of Ti-6Al-4V and Ti-Zr-Nb alloys were studied by Fourier transform infrared spectroscopy (Fig. 2.). Adsorption bands (AB) corresponding to alloy surface groups showed at Fig. 2. AB at 1400–1500 and 871 cm^−1^ indicate stretching vibrations of CO_3_^2−^. AB of metal oxides and metal-OH (depending on alloy nature) bonds on alloys surface appear at 1100–450 cm^−1^.

After coating by HA, all AB corresponding to metal oxide or metal-OH (Me-O) bond vibrations disappear.

### HA-coating in 10×-SBF

**Fig. 3:**
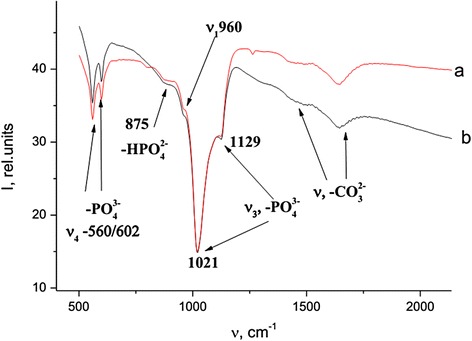
FTIR spectra of Ti-Zr-Nb (*a*) and Ti-6Al-4V (*b*) alloys immersed in 10×-SBF for 24 h

**Fig. 4:**
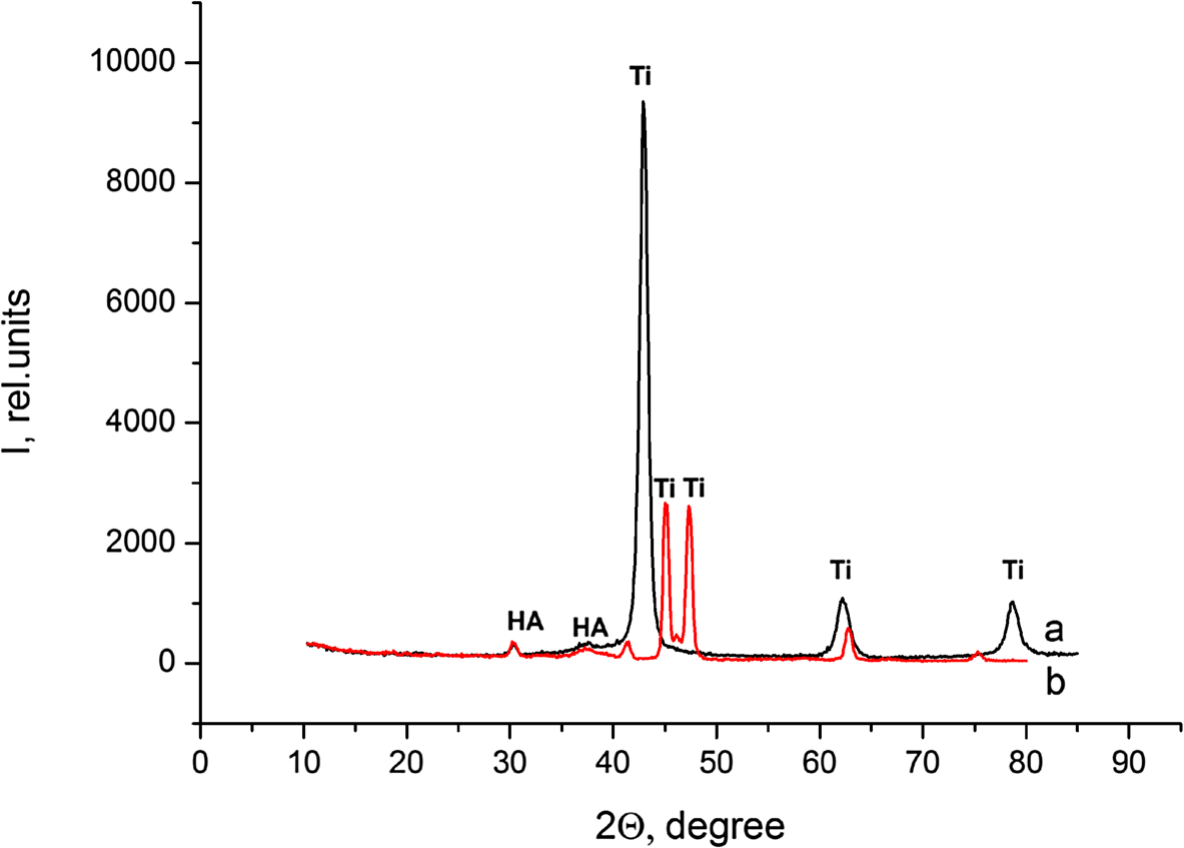
X-ray diffraction patterns of Ti-Zr-Nb (*a*) and Ti-6Al-4V (*b*) alloys immersed in 10×-SBF for 24 h

In the case of 10×-SBF, the HA layer grows no more than 24 h. After that period of time, HA is clearly identifying in IR (Fig. 3) and XRD spectra (Fig. 4).

Generally, FTIR spectra of Ti-Zr-Nb and Ti-6Al-4V alloys immersed in 10×-SBF for 24 h are very close to each other (Fig. 3(a, b)). After 1-day soaking in 10×-concentrated SBF solution, HA is clearly identified in the FTIR spectra.

All the characteristic absorption bands of HA are present. The AB corresponding to *ν*_2_ bending mode of PO_4_^3−^ can be observed at 472 cm^−1^. At 560 and 602 cm^−1^, we can see ν_4_ asymmetric bending vibrations of the PO_4_^3−^ groups. AB at 875 cm^−1^ can be attributed to P–O–H vibration of the HPO_4_^2−^ that is characteristic for Ca-deficient apatite with a deficiency of calcium and nonstoichiometric structure (Ca/P ratio lower than 1.67). Symmetric and asymmetric stretching vibrations of PO_4_^3−^ groups appear at 960 and 1030–1096 cm^−1^, respectively. AB at 1654 cm^−1^ correspond for water bending vibration. A weak carbonate ion peak appears at 1420 and 1496 cm^−1^.

Increasing of the 10×-SBF solution temperature to 80 °C causes an increasing speed of HA precipitation. In those circumstances, HA precipitation occurs within 60 min. Due to good crystallinity of the HA coating obtained in 10×-SBF, diffraction patterns corresponding to HA can be observed on X-ray diffractograms (Fig. 4). The diffraction patterns, ascribed to JCPDS card no. 74-0566 (hydroxyapatite) can be observed at 2*θ* = 13.56, 30.31, and 37.58°. Apparently, process of HA precipitation in warm 10×-SBF solution is connected with the high concentration of the solution and its thermodynamic instability.

**Fig. 5:**
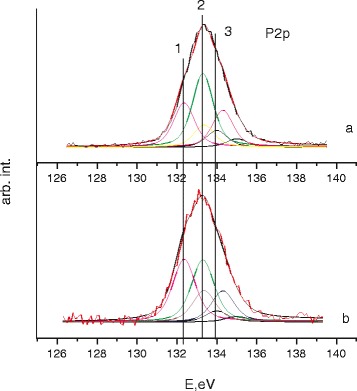
X-ray photoelectron spectra of Р2p levels for TI-6AL-4V (**a**) and Ti-Zr-Nb (**b**) alloys immersed in 10×-SBF

**Fig. 6:**
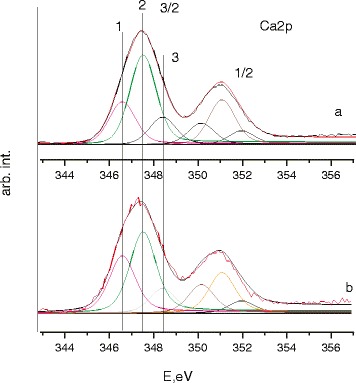
X-ray photoelectron spectra of Са2р levels for TI-6AL-4V (**a**) and Ti-Zr-Nb (**b**) alloys immersed in 10×-SBF

X-ray photoelectron spectra of Са2p and P2p levels were obtained for the samples with different synthesis conditions. The decomposition spectra of the components are shown in Figs. 5 and 6. As we can see from Fig. 5 in TI-6AL-4V (a) and Ti-Zr-Nb (b) samples, phosphorus exists in three nonequivalent conditions. Binding energy varies in the range from 132.4 to 134.0 eV (Р2р_3/2_, component 1). The component with *E*_*b*_ = 132.4 eV corresponds to NaH_2_PO_2_. The component with *E*_*b*_ = 134.0 eV can be attributed to NaPO_3_ whose presence can be explained by nonequilibrium HA precipitation conditions due to oversaturation of simulated body fluid.

The binding energy main component in Са2р_3/2_ spectra in TI-6AL-4V (a) and Ti-Zr-Nb (b) was amounted, *E*_*b*_ = Са2р_3/2_ = 347.5 eV (Fig. 6). Also, the Са2р_3/2_ spectra is present in the low-intensity contribution at *E*_*b*_ = 348.4 eV (component 2), which can be explained by CaCl_2_ signal [20]. Presence of calcium chloride can be explained insufficient washing of the sample.

At *E*_*b*_ = 346.6 eV, we can observe a signal from calcium carbonate (CaCO_3_). It should be noted that the presence of the carbonate ion was also confirmed by FTIR spectroscopy (Fig. 3).

**Fig. 7:**
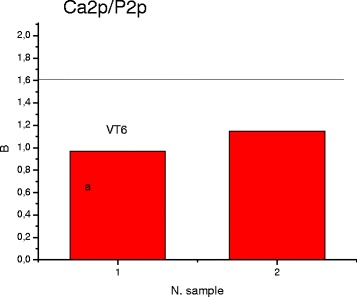
Ca/P ratio in the TI-6AL-4V (*1*) and Ti-Zr-Nb (*2*) alloys, immersed in 10×-SBF

In Fig. 7 the integrated intensity ratios of the Са2р/Р2р levels are given. In the TI-6AL-4V sample, the observed Ca/P ratio reaches 0.97. In the Ti-Zr-Nb sample, the observed Ca/P ratio reaches 1.15.

FTIR spectroscopy previously indicated a deficiency of Ca ions by the presence of AB at 875 cm^−1^ (vibration of the HPO_4_^2−^ that is characteristic for Ca-deficient apatite with deficiency of calcium and nonstoichiometric structure). Despite high efficacy of HA precipitation from an oversaturated SBF solution, XPS data lead us to the conclusion that this process is insufficient in terms of optimal Ca/P ratio.

**Fig. 8:**
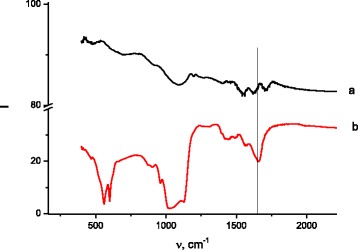
FTIR spectra of Ti-Zr-Nb–COOH (*a*) alloy immersed in 10×-SBF for 24 h (*b*)

**Fig. 9:**
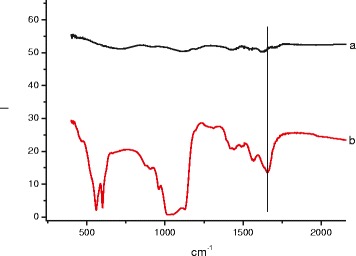
FTIR spectra Ti-6Al-4V–COOH (*a*) alloy immersed in 10×-SBF for 24 h (*b*)

In the case of –COOH-modified surfaces, HA growth take the same time, 24 h. The FTIR spectra for Ti–COOH-modified samples immersed for 24 h in 10×-SBF are shown in Figs. 8 and 9.

Bands of the O–H stretching and bending of H_2_O were seen at, respectively, 3440 and 1649 cm^−1^. The bands at 1490–1420 and 875 cm^−1^ confirmed the presence of carbonate groups. PO_4_ bands were recorded at 570 and 603 (ν_4_), 962 (ν_1_), and 1045 and 1096 (ν_3_) cm^−1^. It is important to note that neither the precipitates themselves nor the coating layer contained CaCO_3_ (calcite) [17–18]. Additional AB at 1430, 1562, and 1660 cm^−1^ (Fig. 9) can be explained by overlapping of –OH, CO_3_^2−^, and –COOH groups. That means, on the surface of –COOH-modified samples, we have HA, carbonated HA, and uncoated carboxyl groups simultaneously.

**Fig. 10:**
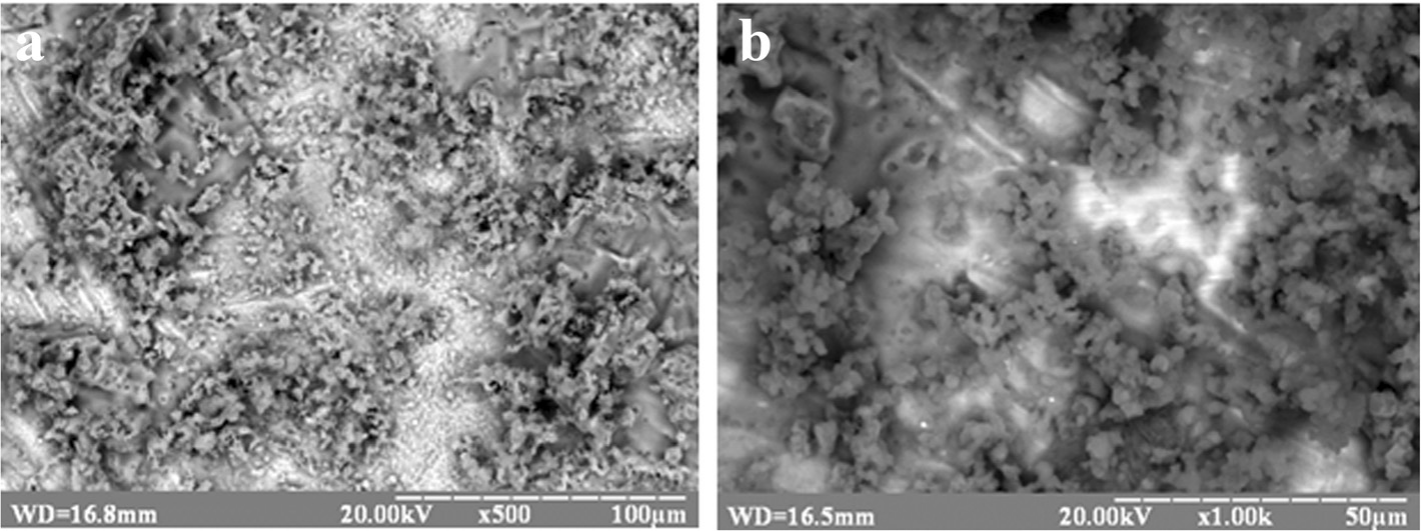
SEM images of Ti-6Al-4V (**a**) and Ti-Zr-Nb (**b**) alloy surfaces coated with HA

SEM images of Ti-6Al-4V–COOH and Ti-Zr-Nb–COOH alloy surfaces after HA deposition are showed on Fig. 10. SEM observations showed that when HA deposited onto Ti-6Al-4V and Ti-Zr-Nb alloy surfaces, it forms irregular structure with HA grain size no more than 50 μm.

As we can conclude from SEM and FTIR data, surface treatment by –COOH group does not have any significant effect on HA formation in 10×-SBF.

## Conclusions

Fourier transform infrared spectroscopy and XRD confirmed the formation of the biomimetic hydroxyapatite coating on the surface of Ti-containing alloys with different quantities of HA as dependent on thermal conditions. By XRD, the influence of thermal conditions on HA crystal formation and size was found. Fourier transform infrared and X-ray photoelectron spectroscopy confirmed that hydroxyapatite with different morphology, crystallinity, and Ca/P ratio formed on metallic surfaces.

In contradistinction to pure Ti, modification of Ti-containing alloy by –COOH groups does not cause any positive effect on HA formation. Coating of Ti-containing alloys by HA can be achieved by simple concentration of SBF and elevating temperature. Despite high efficacy of HA precipitation from oversaturated SBF solution onto the surface of Ti-containing alloys, XPS data lead us to the conclusion that this process is insufficient in terms of optimal Ca/P ratio. X-ray photoelectron spectroscopy showed that in the Ti-6AL-4V sample observed Ca/P ratio reach 0.97, whereas in the Ti-Zr-Nb sample observed Ca/P ratio reach 1.15.

All obtained data show that developed materials are promising for use in medicine as implants with biocompatible surface, similar in composition to natural HA. The results presented in this article may be used to optimize the preparation of biocompatible coatings on titanium and other surfaces.
